# The ability of flagellum-specific *Proteus vulgaris *bacteriophage PV22 to interact with *Campylobacter jejuni *flagella in culture

**DOI:** 10.1186/1743-422X-3-50

**Published:** 2006-06-27

**Authors:** EL Zhilenkov, VM Popova, DV Popov, LY Zavalsky, EA Svetoch, NJ Stern, BS Seal

**Affiliations:** 1 State Research Center for Applied Microbiology, Obolensk, Moscow Region, Russia Federation; 2 Poultry Microbiological Safety Research Unit, Russell Research Center, Agricultural Research Service, USDA, Athens, GA, USA

## Abstract

**Background:**

There has been a recent resurgent interest in bacteriophage biology. Research was initiated to examine *Campylobacter jejuni*-specific bacteriophage in the Russian Federation to develop alternative control measures for this pathogen.

**Results:**

A *C. jejuni *flagellum-specific phage PV22 from *Proteus vulgaris *was identified in sewage drainage. This phage interacted with *C. jejuni *by attachment to flagella followed by translocation of the phage to the polar region of the bacterium up to the point of DNA injection. Electron microscopic examination revealed adsorption of PV22 on *C. jejuni *flagella after a five minute incubation of the phage and bacteria. A different phenomenon was observed after incubating the mix under the same conditions, but for twenty minutes or longer. Phage accumulated primarily on the surface of cells at sites where flagella originated. Interestingly, PV22 did not inject DNA into *C. jejuni *and PV22 did not produce lytic plaques on medium containing *C. jejuni *cells. The constant of velocity for PV22 adsorption on cells was 7 × 10^-9 ^ml/min.

**Conclusion:**

It was demonstrated that a bacteriophage that productively infects *P. vulgaris *was able to bind *C. jejuni *and by a spot test that the growth of *C. jejuni *was reduced relative to control bacteria in the region of phage application. There may be two interesting applications of this effect. First, it may be possible to test phage PV22 as an antimicrobial agent to decrease *C. jejuni *colonization of the chicken intestine. Second, the phage could potentially be utilized for investigating biogenesis of *C. jejuni *flagella.

## Background

*Campylobacter *spp. are commensal bacteria in chickens and can cause a significant proportion of food-borne disease [1]. The high colonization incidences of poultry by campylobacters and the resultant clinical infections in humans have prompted a number of investigations focused upon identifying and subsequently eliminating *Campylobacter *spp. from poultry. Phage typing for *Campylobacter *spp. was developed [2-5] and compared to other classification schemes to trace these bacteria [6]. More recently, the presence of bacteriophage among chickens has been investigated [7,8] along with examining their presence among specified commercial poultry flocks relative to isolates of *C. jejuni *[9]. Dramatic increases in isolation of fluoroquinolone resistant *C. jejuni *have been reported [10] and treatment of chickens with fluoroquinolones can induce rapid selection of ciprofloxacin-resistant campylobacters [11]. Consequently, reduction of *Campylobacter *spp. populations on chicken skin with bacteriophage has been attempted as an alternative control measure to antibiotics with varying degrees of success [7,8,13,14].

There has been a resurgent interest in bacteriophage biology and their use or use of phage gene products as antibacterial agents [15-19]. During ongoing collaborative investigations between our laboratories, a collection of bacteriophages that attach to and/or infect *C. jejuni *were isolated in the Russian Federation to address the issue of utilizing bacteriophage for bacterial control. Interestingly, electron micrographs of a bacteriophage that attaches to *C. jejuni*, but productively infected *Proteus vulgaris *were identified from drainage water samples in the Moscow region. Bacteriophages that infect *P. vulgaris*, as in the case of other bacteria, have been utilized for typing schemes [20-22] and are structurally similar to phage from other bacteria [22-25]. Several of the *Proteus*-phages were shown to attach to the flagella of these bacteria [26,27]. Herein we report the isolation and phage attachment kinetics of a bacteriophage that productively infects *P. vulgaris*, but which attaches to the flagella of *C. jejuni*.

## Results and discussion

**Figure 1:**
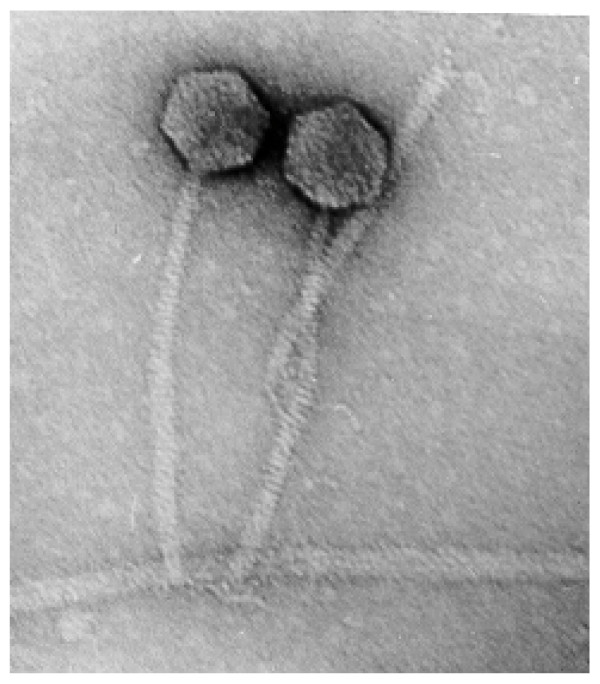
Electron microscopy images of phage PV22 adsorption to *Campylobacter jejuni*. Arrows indicate long flexible tail fibrils the phage utilizes for attachment to *C. jejuni *flagellum; magnification × 200,000.

During research examining bacteriophage from the Moscow region by purifying material from sewage drainage a *C. jejuni *flagellum-specific phage PV22 from *P. vulgaris *was identified (Fig. 1) that structurally most closely resembled members of the *Siphoviridae *[28,29]. The icosohedral head of phage PV22 measured from 56 to 58 nm with a non-contractile tail of greater than 200 nm in length. This phage, PV22, had a wide spectrum of lytic activity to *P. vulgaris *isolates (data not shown), but was subsequently propagated on a single isolate designated 1922. Members of the *Myoviridae*, *Podoviridae *and *Siphoviridae *have been isolated from *P. vulgaris *and utilized as a typing tool for this bacterium [22,25].

**Figure 2:**
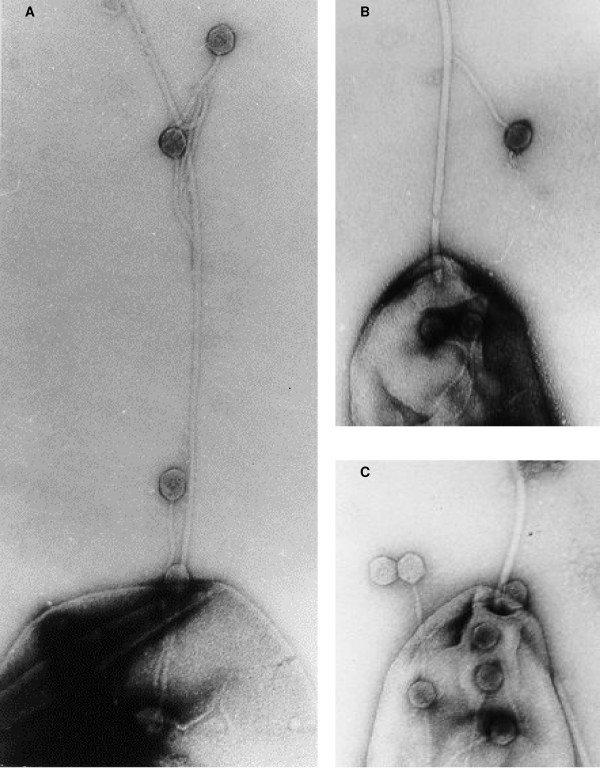
Electron microscopic illustration of phage PV22 interacting with a *Campylobacter jejuni *L4 cell. Phages initially adsorb on the flagellum surface (A) and move toward the cell surface (B) where they accumulate at flagellum origin (C). Magnification is × 50,000.

The adsorption of phage PV22 on the surface of *C. jejuni *flagella was visualized utilizing three different isolates, with the illustration of attachment to *C. jejuni *strain L4 (Fig. 2a, b). Bacteriophage PV22 interacted with *C. jejuni *by attachment followed by translocation of the phage to the polar region of the bacterium up to the point of DNA injection. Electron microscopic examination revealed adsorption of PV22 on *C. jejuni *flagella after a five minute incubation of the phage and bacteria. A different phenomenon was observed when the mix was incubated at the same conditions but for a period of 20 min. or greater. Phage PV22 subsequently accumulated on cell surfaces mainly near areas where flagella originated on *C. jejuni *(Fig. 2c). Interestingly, PV22 did not appear to inject its DNA into *C. jejuni*.

The constant of velocity of PV22 adsorption on cells was determined to be 7 × 10^-9 ^ml/min. Phage PV22 did not produce lytic cells in medium containing *C. jejuni *strains. At the same time, it was demonstrated by a spot test that the growth of *C. jejuni *was reduced relative to control bacteria in the region of phage application. Another observation was that PV22-treated *C. jejuni *cells appeared to lose their capability for chemotaxis (data not shown). Based on preliminary observations it was hypothesized that phage PV22 interacted with H. pylori in a similar manner (data not shown).

Our results suggest that particles of the phage PV22 are interacting with a *C. jejuni *cell on the same lines as infecting a *P. vulgaris *cell wherein certain phage interact by attaching to the flagella [26,27]. However, it should be noted that phage PV22 failed to replicate in *C. jejuni*. Negative results from conventional titration of the phage in the presence of campylobacter cultures provide evidence for this conclusion. Also, phage PV22 did not generate plaque lysis on the surface of lawns produced by *C. jejuni *test cultures. Nevertheless, adsorption of the phage on flagella and in polar areas of the cell may influence *C. jejuni *replication as the cultures had reduced growth within the areas of phage application following spotting on a lawn of *C. jejuni*. It is currently unknown how PV22 fits in the scheme of *Proteus *spp. phage typing [22,25], although structurally it can be classified as a *Siphoviridae *member based on structural characteristics [28,29]. There consequently may be two interesting applications of this effect. First, it may be possible to test phage PV22 as an antimicrobial agent to control *C. jejuni *colonization of the chicken intestine. Second, the phage could potentially be utilized for investigating biogenesis of *Campylobacter jejuni *flagella.

## Methods

### Bacteriophage purification, propagation and bacterial culture

Bacteriophage PV22 was isolated by sampling drainage sewage waters in the Moscow region of the Russian Federation by standard procedures utilizing a *Proteus vulgaris *strain as a host [22,25]. Bacterial cultures of *P. vulgaris *strain 1922 were supplied by the Tarasevich Institute of Standardization and Control of Medicinal Biological Preparations (Moscow) and propagated in meat-peptone broth [21]. Phage PV22was isolated according to the method of Snustad & Dean [30] as described in detail [21] by first clarifying drainage samples by low-speed centrifugation (5,000 × g for 20 min.) followed by filtration of the supernatant through 0.45 and then 0.22 um filters. Resultant filtered supernatants were cultured with *P. vulgaris *strain 1922 for 18 hrs followed by limit-dilution cloning to isolate individual viruses lytic for *P. vulgaris *utilizing standard techniques. *C. jejuni *isolates L4, 11168 and F2 were propagated in Brucella FBP agar and incubated at 42°C for 36–48 hours in microaerobic atmosphere (5% O2, 10% CO2 and 85% N2) as described previously [31].

In order to provide supportive evidence of the interaction between phage PV22 and *C. jejuni*, cultures of phage PV22 were sequentially centrifuged at 7,000 g for 20 min and 30,000 g for 120 min. Pellets obtained were suspended in 0.01 M Tris – HCl buffer (pH 7.0) to 4.75 ml of Tris – HCl buffer, 7 g of CsCl and 0.25 ml of phage suspension were then added to the vial. This was centrifuged in a SW-50 rotor at 35,000 rpm for 48 hours to produce fractions. An aliquot of purified phage was then dialyzed against 0.01 M Tris – HCl buffer (pH 7.0).

### Bacteriophage attachment for electron microscopy and binding assay to *C. jejuni*

Cells of *C. jejuni *were suspended in 0.01 M Tris – HCl buffer (pH 7.0) containing 0.1 M MgSO_4 _and 0.001 M CaCl_2 _were mixed with purified preparation of phage PV22 (MOI of 10) and incubated at 40C in microaerobic conditions for 5 or 20 min. The suspension was centrifuged at 7,000 × g for 5 min., placed onto colloidal supporting films and treated with 1% uranyl acetate for further examination by electron microscopy (Hitachi H-300) utilizing standard methods [32]. The number of phage that bound to *C. jejuni *L4 was determined by first titration of PV22 with *P. vulgaris *strain 1922 and a constant velocity of adsorption was determined by the formula of Adams [33] by titration of the PV22 with its host. A preliminary chemotaxis assay was conducted as described by Adler [34] utilizing a capillary method with chicken epithelial cecal cells as the attractant with phage PV22 at an MOI of 10 with *C. jejuni *or *C. jejuni *alone.

## Competing interests

The author(s) declare that they have no competing interests.

## Authors' contributions

The research was completed at the State Research Center for Applied Microbiology, Obolensk, Russian Federation in the laboratory of E. L. Zhilenkov under the laboratory unit direction of E. A. Svetoch. N. J. Stern is a co-principle investigator for funding with collaborator B. S. Seal at the Poultry Microbiological Safety Research Unit, ARS, USDA in Athens, GA, USA who completed writing and final editing of the manuscript.
